# Statin Therapy Alters Lipid Storage in Diabetic Skeletal Muscle

**DOI:** 10.3389/fendo.2016.00095

**Published:** 2016-07-19

**Authors:** Irena A. Rebalka, Matthew J. Raleigh, Laelie A. Snook, Alexandra N. Rebalka, Rebecca E. K. MacPherson, David C. Wright, Jonathan D. Schertzer, Thomas J. Hawke

**Affiliations:** ^1^Department of Pathology and Molecular Medicine, McMaster University, Hamilton, ON, Canada; ^2^Human Health and Nutritional Sciences, University of Guelph, Guelph, ON, Canada; ^3^Department of Biochemistry and Biomedical Sciences, McMaster University, Hamilton, ON, Canada

**Keywords:** fluvastatin, skeletal muscle, ectopic lipids, FAT/CD36, FABPpm, myopathy

## Abstract

While statins significantly reduce cholesterol levels and thereby reduce the risk of cardiovascular disease, the development of myopathy with statin use is a significant clinical side effect. Recent guidelines recommend increasing inclusion criteria for statin treatment in diabetic individuals; however, the impact of statins on skeletal muscle health in those with diabetes (who already suffer from impairments in muscle health) is ill defined. Here, we investigate the effects of fluvastatin treatment on muscle health in wild type (WT) and streptozotocin (STZ)-induced diabetic mice. WT and STZ-diabetic mice received diet enriched with 600 mg/kg fluvastatin or control chow for 24 days. Muscle morphology, intra and extracellular lipid levels, and lipid transporter content were investigated. Our findings indicate that short-term fluvastatin administration induced a myopathy that was not exacerbated by the presence of STZ-induced diabetes. Fluvastatin significantly increased ectopic lipid deposition within the muscle of STZ-diabetic animals, findings that were not seen with diabetes or statin treatment alone. Consistent with this observation, only fluvastatin-treated diabetic mice downregulated protein expression of lipid transporters FAT/CD36 and FABPpm in their skeletal muscle. No differences in FAT/CD36 or FABPpm mRNA content were observed. Altered lipid compartmentalization resultant of a downregulation in lipid transporter content in STZ-induced diabetic skeletal muscle was apparent in the current investigation. Given the association between ectopic lipid deposition in skeletal muscle and the development of insulin-resistance, our findings highlight the necessity for more thorough investigations into the impact of statins in humans with diabetes.

## Introduction

Statins inhibit 3-hydroxy-3-methylglutaryl-CoA reductase, thereby inhibiting cholesterol biosynthesis and LDL formation with great efficacy. As reductions in circulating LDL cholesterol levels greatly reduce atherosclerotic cardiovascular disease (ASCVD) risk, statins are among the most widely prescribed pharmaceuticals in the world (1–3). While considerable evidence supports the benefits of these cholesterol-lowering drugs in reducing ASCVD risk, statin therapies have also been associated with serious side effects, such as myopathy. Recent reports estimate up to 25% of statin users experience some form of myopathic/myalgia symptoms, ranging from muscle soreness to severe rhabdomyolysis (4–6). Despite these concerns, the American College of Cardiology (ACC) and American Heart Association (AHA) 2013 guidelines recommend that all diabetic individuals over the age of 40 be prescribed statins regardless of their ASCVD risk (5).

Muscle from those with Type 1 diabetes mellitus (T1DM) exhibits myopathic features; a complication termed diabetic myopathy (7). Decreases in muscle mass (8) and reduced myofiber size (9), translating to muscle weakness and reduced physical capacity (10, 11) have all been shown in individuals with T1DM. While new statin therapy guidelines were implemented to reduce ASCVD risk in diabetic individuals, it has not yet been elucidated if the combination of T1DM and statin administration exacerbates the myopathy resultant from each factor alone. To that end, we fed wild-type (WT) and streptozotocin (STZ)-treated mice (T1DM mouse model) a control or fluvastatin-enriched diet for a period of 24 days and harvested their skeletal muscle to assess metabolic and morphologic characteristics.

## Materials and Methods

### Animal Handling and Tissue Collection

Experimentation was approved by the McMaster University Animal Research Ethics Board, in accordance with the Canadian Council for Animal Care guidelines. At 10–12 weeks of age, male C57BL6/J mice (Jackson Laboratories, Bar Harbor, ME, USA) were randomly assigned into WT or STZ-diabetic [single 150 mg/kg intraperitoneal STZ injection (Calbiochem, San Diego, CA, USA)] groups. Six weeks after diabetes onset (blood glucose >14 mM), animals were further subdivided and assigned to receive either diet enriched with 600 mg/kg fluvastatin or control chow [D12081101, D12450K, respectively; OpenSource Diets, New Brunswick, NJ, USA]. Diet was administered *ad libitum* for 24 days, after which all animals were euthanized, and tissues were embedded and/or snap frozen for later analyses.

### Histochemical and Immunofluorescent Analysis

Frozen TA (tibialis anterior) muscle sections were stained *via* hematoxylin–eosin (H&E) to quantify centrally located nuclei, necrotic fibers, and myofiber areas. Laminin and dystrophin (both 1:250; Abcam, Cambridge, UK) fluorescent co-stain was used to determine the number of split myofibers. Oil Red O (ORO) staining was used to quantify intramyocellular lipid density. Analysis of perilipin (1:200; Cell Signaling, Danvers, MA, USA) was used for determination of ectopic lipid droplet number and size per unit area. All imaging and analysis was undertaken on a Nikon 90i microscope using Nikon NIS-Elements ND2 software (Melville, NY, USA).

### Western Blotting

Gastrocnemius, plantaris, and soleus (GPS) muscle was homogenized in NP40 Lysis Buffer supplemented with phenylmethylsulfonylfluoride and Protease Inhibitor Cocktail. Western blotting was undertaken as previously described (12) using anti-FAT/CD36 (Santa Cruz, Dallas, TX, USA) and anti-FABPpm (generous gift from J. Calles-Escandon, Wake Forest University, NC, USA) and primary antibodies and appropriate horseradish peroxidase-conjugated secondary antibodies. Bands were quantified *via* densitometry (Alpha Innotech Fluorchem HD2, ThermoFisher Scientific, Waltham, MA, USA) with equal loading confirmed by PonceauS staining (Sigma-Aldrich, St. Louis, MO, USA).

### Real-time PCR

Total RNA was extracted from GPS using Trizol reagent and reversed transcribed into cDNA. Changes in mRNA expression were determined using real-time qPCR and Taqman gene expression assays for mouse CD36 (Mm_00432403_m1), FABPpm (Mm00494703_m1), and GAPDH (4352932E) (Applied Biosystems, Foster City, CA, USA) as previously described in Ref. (13).

### Statistics

All statistical analyses were performed using Prism 6 (GraphPad Software, La Jolla, CA, USA). Statistical significance was determined *via* unpaired *t*-test, and defined as *p* ≤ 0.05.

## Results

### Analysis of Myopathy

**Figure 1:**
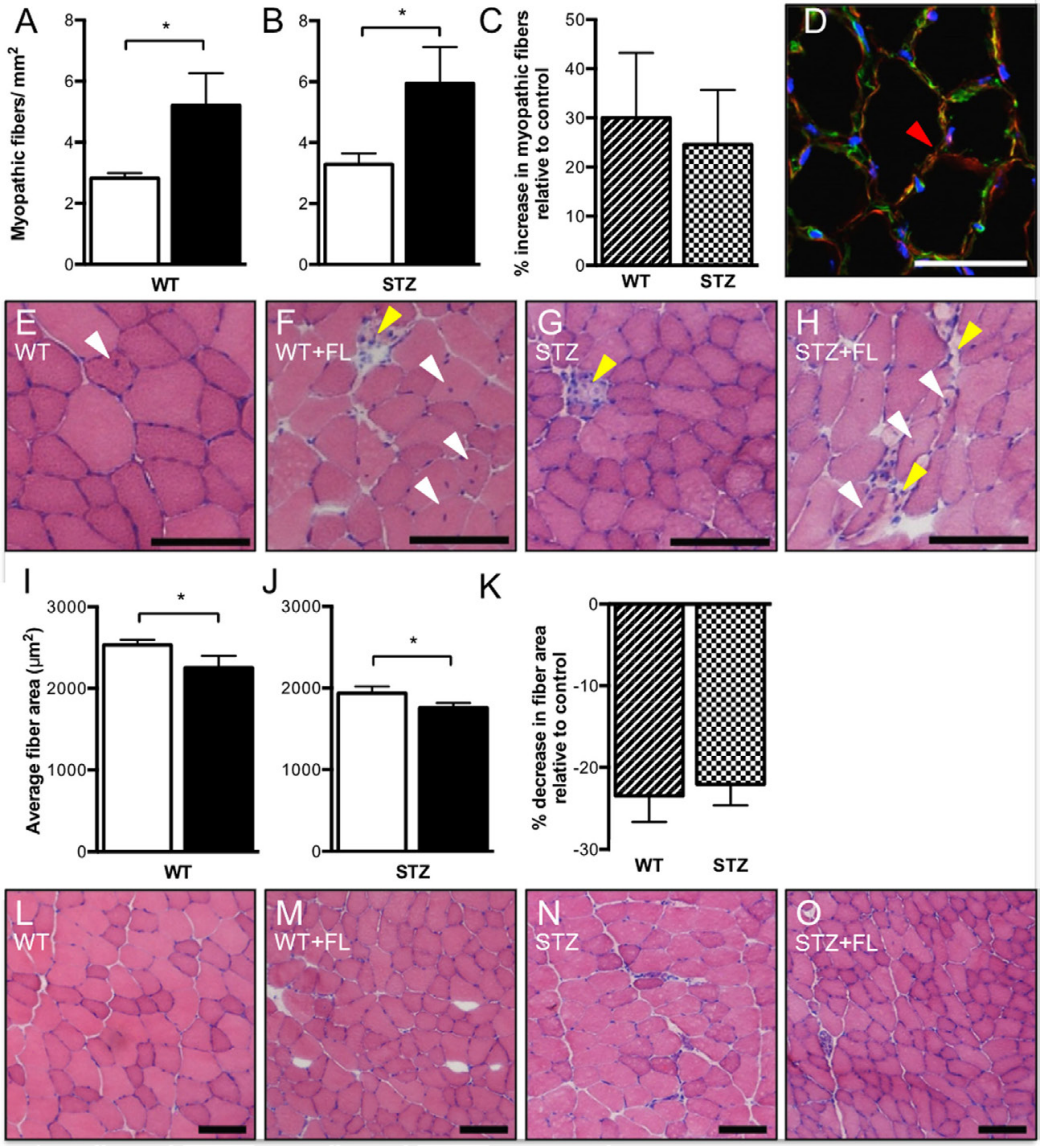
**Short-term fluvastatin administration causes hallmark phenotypes of myopathy**. No differences in severity of myopathy, however, are noted between WT- and STZ-diabetic skeletal muscle. When compared to their control treated counterparts, fluvastatin administration results in an increased amount of total myopathic fibers in both WT **(A)** and STZ **(B)** muscle. No differences in severity of myopathy as a result of fluvastatin administration, however, are noted between WT and STZ skeletal muscle (*p* = 0.38) **(C)**. A red arrowhead, indicating an example of a split fiber, is shown in **(D)**. Representative myopathic images for each group are shown in **(E–H)**. White arrowheads indicate centrally nucleated fibers, while yellow arrowheads indicate necrotic fibers. A decrease in average fiber area as a result of fluvastatin administration is noted in both WT **(I)** and STZ **(J)** muscle, and, once again, no differences in the degree of this decrease are noted between WT and STZ muscle (*p* = 0.37) **(K)**. Images depicting representative fiber size for each group are shown in **(L–O)**. For **(A,B,I,J)**, white bars indicate control treatment, and black bars indicate fluvastatin treatment. For **(C,K)**, Striped bars indicate percent change in fluvastatin-treated WT muscle relative to control-treated WT muscle, and checked bars indicate percent change in fluvastatin-treated STZ muscle relative to control-treated STZ muscle. Scale bar in **D** = 50 μm. Scale bar in **(E–H,L–O)** = 100 μm. *Significant differences (*p* ≤ 0.05). All data presented as mean ± SEM. *n* = 5–6 for each bar in **(A–C)**. *n* = 4–6 for each bar in **(I–K)**.

Centrally nucleated, necrotic and split myofibers were evaluated and summated as “myopathic fibers” to characterize myopathy. Fluvastatin administration increased total myopathic fibers in TA muscle from both WT (Figure 1A) and STZ-treated (Figure 1B) mice. Representative images are shown in Figures 1D–H. Although myopathy was observed in both WT and STZ muscle as a result of fluvastatin administration, no difference in the severity of myopathy was noted between WT and STZ muscle (Figure 1C). When compared to control-treated muscle, fiber cross-sectional area was significantly reduced following fluvastatin treatment in both WT (Figure 1I) and STZ (Figure 1J) muscle, supporting a greater presence of atrophied, myopathic fibers. Representative images are shown in Figures 1L–O. Once again, no differences in myofiber area were noted between WT and STZ muscle as a result of fluvastatin treatment (Figure 1K).

### Extracellular and Intramyocellular Lipid Analysis

**Figure 2:**
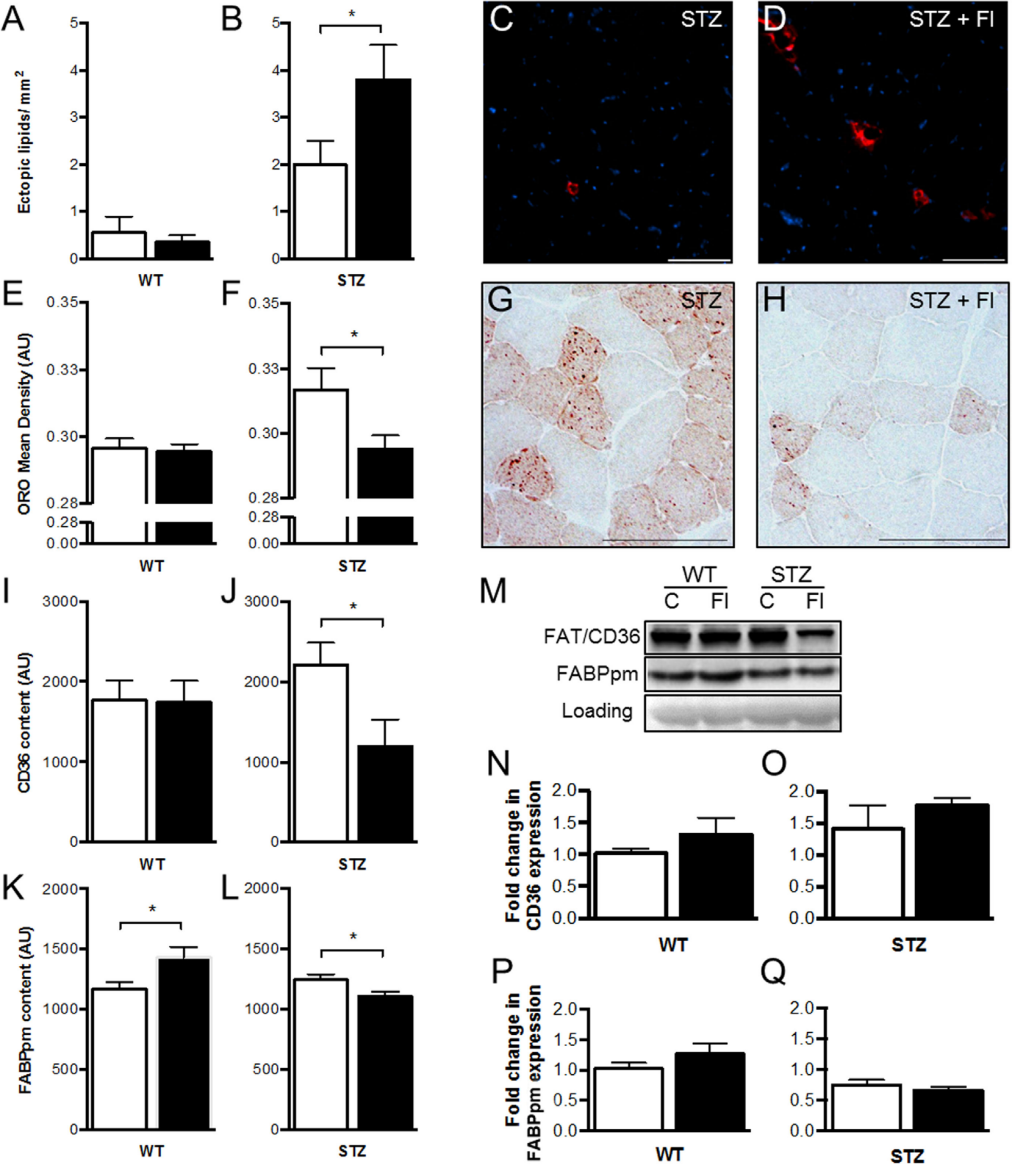
**Short-term fluvastatin administration affects lipid and fatty acid transporter content in STZ-diabetic skeletal muscle**. Whereas fluvastatin administration causes no modifications in ectopic lipid content of WT muscle (*p* = 0.31) **(A)**, STZ muscle displays significant increases in ectopic lipid content [and ectopic lipid size (*p* < 0.05)] as a result of fluvastatin administration **(B)**. Representative perilipin-stained (ectopic lipid) images of STZ control and fluvastatin-treated STZ (STZ + Fl) muscle are depicted in **(C,D)**, respectively. Oil Red O (ORO) analysis, once again, reveals no differences in WT muscle IMCL content as a result of fluvastatin administration (*p* = 0.41) **(E)**. STZ muscle, however, **(F)** displays significant decreases in IMCL density in the presence of fluvastatin. Representative IMCL images of STZ control and fluvastatin-treated STZ muscle are depicted in **(G,H)**, respectively. No differences in FAT/CD36 content were apparent in WT muscle as a result of fluvastatin administration (*p* = 0.47) **(I)**, while STZ muscle displayed decreases in FAT/CD36 content with treatment **(J)**. FABPpm, contrastingly, was elevated in WT muscle as a result of fluvastatin administration **(K)**, and, once more, STZ muscle displayed decreases in FABPpm content with fluvastatin treatment **(L)**. A representative immunoblot, including loading control (PonceauS), is shown in **(M)**. No differences in FAT/CD36 or FABPpm mRNA expression were observed with treatment **(N–Q)**; graphs depicted as fold-change relative to respective control. White bars indicate control treatment, black bars indicate fluvastatin treatment. *Significant differences (*p* < 0.05). All data presented as mean ± SEM. *n* = 5–6 for each bar in **(A–Q)**. Scale bar = 100 μm in **(C,D,G,H)**.

Histological examination by H&E staining revealed transparent globules adjacent to muscle fibers that appeared to be ectopic lipid deposits, most notably in fluvastatin-treated groups. To verify this hypothesis, perilipin (member of a family of proteins that associate with the surface of lipid droplets/adipocytes) analysis was conducted. Quantification demonstrated a significant elevation in ectopic lipid content and size within STZ-diabetic muscle following fluvastatin administration, an effect that was not observed in other groups (Figures 2A–D).

With respect to intramyocellular lipid droplets (IMCLs), no differences in content were evident in WT muscle with treatment (Figure 2E). In contrast, fluvastatin-treated STZ-diabetic mice exhibited significantly reduced IMCL content relative to their diabetic counterparts (Figures 2F–H).

### Lipid Transporter Analysis

When compared to control-treated muscle, no differences in FAT/CD36 content were apparent in WT muscle as a result of fluvastatin administration (Figure 2I), while STZ-diabetic muscles displayed decreases in FAT/CD36 content with fluvastatin treatment (Figure 2J). FABPpm analysis revealed elevations in protein content in WT muscle as a result of fluvastatin administration (Figure 2K), and, mirroring FAT/CD36 analysis, decreases in FABPpm content in fluvastatin-treated STZ muscle (Figure 2L).

In order to ascertain if fluvastatin treatment was affecting the transcriptional regulation of lipid transporters, mRNA analysis was conducted. No differences in FAT/CD36 or FABPpm mRNA content were observed between groups (Figures 2M–Q).

## Discussion

Recent ACC/AHA guidelines recommend the widespread use of statin therapy as a primary means to reduce ASCVD risk in all persons over 40 years old with diabetes mellitus (5). While statin therapy lowers ASCVD risk, these HMG-CoA reductase inhibitors are also associated with a risk of myotoxicity (4), and their impact on skeletal muscle health in those with T1DM has not yet been fully examined. Many rodent studies investigating the impact of statins on myopathy have utilized long-term administration or statins with a stronger myotoxic profile (14). The present study, however, utilized short-term exposure of fluvastatin; a statin generally agreed to have a low clinical risk of myotoxicity (15). Despite a low myotoxic profile, fluvastatin has been reported to activate the NLRP3 inflammasome and induce adipose tissue insulin resistance in obese animals at levels comparable to, or greater than lovastatin, atorvastatin, and simvastatin (16).

Here, we report that short-term fluvastatin administration resulted in a myopathic phenotype, the characteristics of which were not exacerbated with STZ-induced diabetes. Statin-treated diabetic mice did however demonstrate a significant increase in the presence of skeletal muscle ectopic lipid droplets, an observation that was not present in statin-treated, non-diabetic mice. In support of this observation, statin-treated diabetic mice displayed significant decreases in lipid transporter content within their skeletal muscle. These findings raise the possibility that statin administration in T1DM humans could contribute to the impairments in skeletal muscle health, including insulin resistance (17).

Type 1 diabetes mellitus negatively impacts skeletal muscle health, a complication referred to as diabetic myopathy (7). Here, we chose an early time point of T1DM progression, where certain myopathic characteristics (centrally located nuclei, split and necrotic fibers) would be minimal, thus allowing us to assess if statin treatment exacerbated the progression of diabetic myopathy. In contrast to our hypothesis, statin-treated mice did not display a worsened myopathy in the presence of diabetes. Future studies using a more established diabetic myopathy, or a statin with a stronger myotoxicity profile [e.g., simvastatin (15)], would address this question more fully.

While we hypothesized that statin administration would worsen the quality of diabetic muscle, the profound effects of statin treatment on ectopic lipid droplet formation within diabetic muscle was an unexpected and, to our knowledge, novel finding. Muscles of T1DM mice have elevated IMCL content with a concomitant increase in lipid transporters [present study, (12)]. Interestingly, when diabetic mice are on statin therapy, a significant rise in lipid content outside of the myofibers was noted without an increase in IMCLs, indicating potential complications with myofiber lipid transport. Supportive of this altered lipid compartmentalization, interrogation of lipid transporters demonstrated that FAT/CD36 and FABPpm protein content was significantly downregulated in statin-treated diabetic skeletal muscle. FAT/CD36 and FABPpm are integral membrane proteins that, following the appropriate stimulus, are mobilized from their intracellular pools to be integrated into the plasma membrane (18). As mRNA content of these proteins was not different between groups, these findings suggest the observed changes in lipid transporter content to be post-translational in nature. Specifically, the observed lipidopathy is hypothesized to be a result of attenuated transporter palmitoylation, impairing transporter mobilization and ultimate metabolic functionality. Indeed, previous work has implicated statins in the reduction of palmitoylation (19) as well as in the impairment of protein trafficking and localization of FAT/CD36 (20).

Elevated lipid transporter content is important in the muscles of poorly controlled Type 1 diabetic individuals to provide an adequate fuel supply in the absence of insulin (and resultant lack of glucose uptake). The present data would suggest that the increased lipid availability observed in diabetes coupled with the statin-mediated decreases in lipid transporter availability would hinder lipid uptake and result in an accumulation of ectopic lipids within the diabetic muscle. Given the association between skeletal muscle ectopic lipids and the development of insulin resistance, the adverse effects of statin administration in the presence of overt diabetes could have profound effects on glycemic control in those with insulin-dependent diabetes mellitus.

Future studies should focus on identifying whether statin treatment in humans with diabetes decreases lipid transporter availability and, in turn, increases the presence of ectopic lipids within skeletal muscle. Furthermore, to complement the findings of this investigation, future studies should investigate the exacerbation of lipid deposition in other tissues (heart and liver) as lipid accumulation in these tissues has been linked to dysfunction and insulin resistance (21).

## Author Contributions

IR, JS, DW, and TH designed the experiments. IR, MR, LS, AR, and RM performed experiments and data analyses. IR and TH wrote the manuscript. All authors revised the manuscript and provided final approval of the version to be published.

## Conflict of Interest Statement

The authors declare that the research was conducted in the absence of any commercial or financial relationships that could be construed as a potential conflict of interest.

## Abbreviations

ACC, American College of Cardiology; AHA, American Heart Association; ASCVD, atherosclerotic cardiovascular disease; GPS, gastrocnemius, plantaris, soleus; H&E, hematoxylin–eosin; IMCL, intramyocellular lipid; ORO, oil red O; STZ, streptozotocin; T1DM, type 1 diabetes mellitus; TA, tibialis anterior; WT, wild-type.
